# Supplementary material for: Dysregulated Retinoic Acid Signaling in the Pathogenesis of Pseudoexfoliation Syndrome
Source: Int J Mol Sci. 2022 May 26;23(11):5977. doi: 10.3390/ijms23115977 (PMC9180992; doi:10.3390/ijms23115977)

Table S1: Differentially expressed genes in iris tissue of PEX patients as compared to normal donors. PCR-Array analysis (n=3). Verification by means of specific qRT-PCR primer assays (n=24). Asterisks indicate statistical significance (*p<0.05, **p<0.01, ***p<0.001; nd, not detected).

Gene symbol	Gene name	Fold up- / downregulation (mean ± SD)	
		RT² Profiler PCR array (n=3)	qRT-PCR primer assays (n=24)
ADH1	Alcohol dehydrogenase 1A (class I), alpha polypeptide	-1.58 ± 0.79	-1.57 ± 0.61***
ALDH1A1	Aldehyde dehydrogenase 1 family, member A1	-1.83 ± 0.15*	-1.93 ± 0.92***
ALDH1A2	Aldehyde dehydrogenase 1 family, member A2	1.47 ± 0.14	-1.10 ± 0.58
ALDH1A3	Aldehyde dehydrogenase 1 family, member A3	1.1 ± 0.68	1.11 ± 0.77
APOA2	Apolipoprotein A-II	1.55 ± 0.49	
ASCL1	Achaete-scute complex homolog 1 (Drosophila)	1.06 ± 0.76	
BHLHE40	Basic helix-loop-helix family, member e40	1.45 ± 0.36	
BMP2	Bone morphogenetic protein 2	-1.68 ± 0.62	
CD38	CD38 molecule	1.99 ± 0.17*	
CDX1	Caudal type homeobox 1	1.76 ± 0.72	
CHD7	Chromodomain helicase DNA binding protein 7	-1.39 ± 0.49	
CRABP1	Cellular retinoic acid binding protein 1	1.11 ± 1.07	-1.50 ± 1.17*
CRABP2	Cellular retinoic acid binding protein 2	-1.66 ± 1.39	-1.41 ± 0.64*
CYP1B1	Cytochrome P450, family 1, subfamily B, polypeptide 1	-1.48 ± 0.21	
CYP26A1	Cytochrome P450, family 26, subfamily A, polypeptide 1	1.03 ± 0.84	
CYP26B1	Cytochrome P450, family 26, subfamily B, polypeptide 1	-1.32 ± 1.34	
CYP26C1	Cytochrome P450, family 26, subfamily C, polypeptide 1	1.59 ± 0.80	
DCX	Doublecortin	nd	
DHRS3	Dehydrogenase/reductase (SDR family) member 3	-2.17 ± 0.83	
DHRS9	Dehydrogenase/reductase (SDR family) member 9	1.16 ± 0.59	
DLX5	Distal-less homeobox 5	1.63 ± 0.83	
EFNB1	Ephrin-B1	1.22 ± 0.57	
EGR1	Early growth response 1	2.42 ± 0.77	
EPO	Erythropoietin	2.72 ± 3.05	
FABP5	Fatty acid binding protein 5 (psoriasis-associated)	-1.15 ± 0.21	
FGF8	Fibroblast growth factor 8 (androgen-induced)	-1.04 ± 0.47	
FOXA1	Forkhead box A1	-1.13 ± 0.21	
FOXG1	Forkhead box G1	-1.72 ± 0.64	

GATA4	GATA binding protein 4	-1.06 ± 1.05	
GBX2	Gastrulation brain homeobox 2	3.03 ± 1.15	
GLI1	GLI family zinc finger 1	-1.02 ± 0.11	
HNF1B	HNF1 homeobox B	1.71 ± 1.62	
HOXA1	Homeobox A1	2.11 ± 0.71	
HOXA5	Homeobox A5	1.67 ± 0.70	
HOXB1	Homeobox B1	1.28 ± 0.38	
HOXB4	Homeobox B4	2.84 ± 1.51	
HSD17B2	Hydroxysteroid (17-beta) dehydrogenase 2	-4.42 ± 2.1*	-2.19 ± 0.88***
ISL1	ISL LIM homeobox 1	-1.03 ± 0.81	
JAG1	Jagged 1	1.12 ± 0.71	
KLF4	Kruppel-like factor 4 (gut)	2.54 ± 0.86	
LEFTY1	Left-right determination factor 1	-1.04 ± 0.66	
LHX1	LIM homeobox 1	1.24 ± 1.17	
LRAT	Lecithin retinol acyltransferase	1.47 ± 0.87	-1.17 ± 0.82
MAFB	V-maf musculoaponeurotic fibrosarcoma oncogene homolog B (avian)	1.71 ± 0.91	
MEIS2	Meis homeobox 2	-1.8 ± 0.66	
MSX2	Msh homeobox 2	1.28 ± 0.92	
MYC	V-myc myelocytomatosis viral oncogene homolog (avian)	1.13 ± 0.83	
NANOG	Nanog homeobox	2.03 ± 0.74	
NEUROD1	Neurogenic differentiation 1	1.13 ± 0.96	
NRIP1	Nuclear receptor interacting protein 1	-1.45 ± 0.27	
OLIG2	Oligodendrocyte lineage transcription factor 2	1.87 ± 0.76	
OTX2	Orthodenticle homeobox 2	-2.98 ± 1.93	-2.10 ± 1.23***
PAX6	Paired box 6	-1.49 ± 0.11*	
PITX2	Paired-like homeodomain 2	-1.69 ± 1.04	
PLAT	Plasminogen activator, tissue	2.51 ± 0.95	
PPARA	Peroxisome proliferator-activated receptor alpha	-1.97 ± 0.35	-1.13 ± 0.51
PPARD	Peroxisome proliferator-activated receptor delta	-1.84 ± 0.59*	-1.06 ± 0.42
PPARG	Peroxisome proliferator-activated receptor gamma	-1.68 ± 0.45	-2.19 ± 1.30***
RARA	Retinoic acid receptor, alpha	-1.26 ± 0.21	-1.55 ± 0.69***
RARB	Retinoic acid receptor, beta	-1.80 ± 0.41**	-2.37 ± 1.23***
RARG	Retinoic acid receptor, gamma	1.05 ± 0.81	-1.09 ± 0.43
RARRES3	Retinoic acid receptor responder (tazarotene induced) 3	1.18 ± 0.23	
RBP1	Retinol binding protein 1, cellular	-1.36 ± 0.38	-1.48 ± 0.56**

RBP2	Retinol binding protein 2, cellular	-1.46 ± 0.64	-1.67 ± 0.81***
RBP4	Retinol binding protein 4, plasma	-1.51 ± 0.88	-1.46 ± 0.59**
RDH10	Retinol dehydrogenase 10 (all-trans)	-1.65 ± 0.75	-1.66 ± 0.59***
RET	Ret proto-oncogene	-1.49 ± 0.97	
RXRA	Retinoid X receptor, alpha	-1.26 ± 0.29	-1.56 ± 0.71*
RXRB	Retinoid X receptor, beta	-1.19 ± 0.47	-1.10 ± 0.35
RXRG	Retinoid X receptor, gamma	2.30 ± 1.47	1.77 ± 0.57***
SHH	Sonic hedgehog	-2.14 ± 1.33	
SOX2	SRY (sex determining region Y)-box 2	-3.39 ± 0.58*	-1.57 ± 1.26*
SOX9	SRY (sex determining region Y)-box 9	1.06 ± 0.31	
SREBF1	Sterol regulatory element binding transcription factor 1	2.58 ± 1.42	-1.44 ± 0.56**
STRA6	Stimulated by retinoic acid gene 6 homolog (mouse)	-2.00 ± 1.15	-1.82 ± 0.96***
STRA8	Stimulated by retinoic acid gene 8 homolog (mouse)	-1.75 ± 0.50	
TBX1	T-box 1	-2.99 ± 1.37	
TFAP2C	Transcription factor AP-2 gamma	-2.28 ± 0.47	
TGFB2	Transforming growth factor, beta 2	-1.89 ± 0.27**	
TGM2	Transglutaminase 2	-1.27 ± 0.48	
TUBB3	Tubulin, beta 3	4.64 ± 2.57	
UCP1	Uncoupling protein 1 (mitochondrial, proton carrier)	1.11 ± 0.51	
WNT5A	Wingless-type MMTV integration site family, member 5A	-2.88 ± 0.76*	
WNT8A	Wingless-type MMTV integration site family, member 8A	-3.9 ± 1.84	-1.57 ± 0.61***

Table S2: Differentially expressed genes of the retinoic acid signaling pathway in iris, ciliary body, retina and choroid of PEX patients (n=23) as compared to normal donors (n=23) analyzed by real-time PCR. Data are given as fold change of expression in PEX as compared to control samples (*p<0.05, **p<0.01, ***p<0.001; nd, not detected).

		Fold change (mean)			
**Gene symbol**	**Gene name**	**Iris**	**Ciliary body**	**Retina**	**Choroid**
**ADH1**	Alcohol dehydrogenase 1A (class I), alpha	0.64***	0.74*	0.64*	0.60**
**ADH2**	Alcohol dehydrogenase 1B (class I), beta	0.82	1.11	1.09	0.96
**ADH3**	Alcohol dehydrogenase 1C (class I), gamma	1.07	1.05	0.95	1.13
**ADH4**	Alcohol dehydrogenase 4 (class II), pi	nd	nd	nd	nd
**ADH5**	Alcohol dehydrogenase 5 (class III), chi	1.12	1.08	0.97	1.03
**ALDH1A1**	Aldehyde dehydrogenase 1 family, member A1	0.52***	0.62***	0.62*	0.69*
**ALDH1A2**	Aldehyde dehydrogenase 1 family, member A2	0.91	1.19	1.02	0.90
**ALDH1A3**	Aldehyde dehydrogenase 1 family, member A3	1.11	1.01	0.93	1.21
**ALDH8A1**	Aldehyde dehydrogenase 8 family member A1	0.75	0.80	0.49***	0.62***
**CRABP1**	Cellular retinoic acid binding protein 1	0.67*	0.66*	0.53**	0.73**
**CRABP2**	Cellular retinoic acid binding protein 2	0.71*	0.54***	0.57*	0.55*
**CYP26A1**	Cytochrome P450, family 26, subfamily A1	nd	nd	nd	nd
**CYP26B1**	Cytochrome P450, family 26, subfamily B1	0.92	0.92	1.17	0.98
**CYP26C1**	Cytochrome P450, family 26, subfamily C1	nd	nd	nd	nd
**LRAT**	Lecithin retinol acyltransferase	0.86	1.35	0.91	1.02
**RARA**	Retinoic acid receptor, alpha	0.65***	0.69***	0.70	1.24
**RARB**	Retinoic acid receptor, beta	0.42***	0.70*	0.62**	0.69*
**RARG**	Retinoic acid receptor, gamma	0.91	1.08	1.19	0.98
**RBP1**	Retinol binding protein 1, cellular	0.67**	0.65**	0.72	0.71**
**RBP2**	Retinol binding protein 2, cellular	0.60***	0.61***	0.67**	0.66***
**RBP3**	Retinol binding protein 2, interstitial	1.18	1.23	1.13	0.96
**RBP4**	Retinol binding protein 4, plasma	0.68**	0.68**	0.62**	0.52**
**RDH5**	Retinol dehydrogenase 5	1.07	1.14	0.89	1.14
**RDH10**	Retinol dehydrogenase 10	0.60***	0.68*	0.59*	0.77
**RORA**	RAR related orphan receptor A	0.66**	0.81**	0.41***	0.87
**RORB**	RAR related orphan receptor B	0.85	0.83	0.59**	1.20
**RORC**	RAR related orphan receptor C	0.51***	0.69*	0.61**	0.69*
**RXRA**	Retinoid X receptor, alpha	0.64*	0.74*	0.64*	0.68*
**RXRB**	Retinoid X receptor, beta	0.91	0.93	0.76*	0.73*
**RXRG**	Retinoid X receptor, gamma	1.77***	1.52**	0.92	1.23
**STRA6**	Signaling receptor and transporter of retinol STRA6	0.55***	0.75***	0.60*	0.81
**TTR**	Transthyretin	0.88	1.11	0.92	1.22

Table S3. SYBR green primers used for qRT-PCR (T_{an}, annealing temperature).

Gene symbol	Accession	Product length	T_{an}	$MgCl_2$	Sequence 5' - 3'
ADH1	NM_000667.4	198	61	3.0	TCACCAGTCTCCTGGTCTGC<br>CCACGTGGTCATCTGTGCC
ADH2	NM_000668.6	148	62	3.0	ATCAGCTGGGGAATTGAAGCCA<br>AGGGTCCCCTCCACTGGGAT
ADH3	NM_000669.5	167	61	3.0	TGGACGCACGTGGAAAGGAG<br>TTCCAGAGCGAAGCAGGTCA
ADH4	NM_000670.4	270	62	3.0	CACGGAACTCCCTGGGTAGG<br>AGGCGATGGCTGCTTTGCATT
ADH5	NM_000671.4	258	64	3.5	GGGCTGTAAAGTGGCTGGTGC<br>GCTACTCCAACCACGACGCTG
ALDH1A1	NM_000689.5	209	63	3.0	TGCGCATTGCCAAAGAGGAGA<br>CTGGGCACTTACCACGCCAT
ALDH1A2	NM_003888.4	195	62	3.0	TCCAGCAAGATAGAGATGCCCG<br>ACACACCTGTTCTCCTGTGGCT
ALDH1A3	NM_000693.4	212	62	3.0	TAAGCCCGACGTGGACAAGG<br>TACAGCCCTCCAGGTCGATGA
ALDH8A1	NM_170771.2	177	59	3.0	CAGACAGTGGTGGGGACAAC<br>TGCAATACACTTCCCCTGTTGA
COL1A1	NM_000088.3	127	62	3.0	CGAAGACATCCCACCAATCACC<br>GATCACGTCATCGCACAACACC
COL3A1	NM_000090.3	239	64	3.0	GGCTACTTCTCGCTCTGCTTCATC<br>TGGGCAAACTGCACAACATTCTCC
COL4A1	NM_001845.6	116	63	3.0	CAGAGATGGTGTTGCAGGAGTG<br>TGTCACCTTTGAGCCGCAAGTC
CRABP1	NM_004378.3	270	62	3.0	AGGTCGGAGAAGGCTTTGAGGA<br>TATTCCTCAGGGGAGCCTGCAT
CRABP2	NM_001878.4	163	59	3.0	CTCCTCCCCAACCTTGAAGT<br>GGAATTGCTCAAAGTGCTGG
CTGF	NM_001901.4	250	62	3.0	CCTGGAAGAGAACATTAAGAAGGGCA<br>GTTGTAATGGCAGGCACAGGTC
CYP26A1	NM_000671.4	102	64	3.5	GCTGCGATCAAGCTCTGGGAC<br>TTCCCCAAAGAAGGGGAAGCC
CYP26B1	NM_019885.3	249	63	3.0	CGCAAGATCCTCATGGGCGAG<br>CGCCTCCTGGTACACGTTGA
CYP26C1	NM_183374.3	91<br>102	64	3.5	ACGGGCTGCGGCTCTTTTTC<br>AAGCCGTGTCCTAGAGCGCA
ELN	NM_000501.2	197	62	3.0	AGGCAAACCTCTTAAGCCAGTTCC<br>CAGACACTCCTAAGCCACCAACTC
FBLN1	NM_006487.3	231	62	3.5	CTGCGAGTACAGCCTCATGG<br>AAGCAGGAGCAGACCACCTC
FBLN2	NM_001998.3	78	65	4.5	AGGACACAGACCCCAACTCTGTC<br>AAGTCTCGATCAGGTCCTTGGTG
FBLN3	NM_001039349.3	245	62	3.5	TGAACAGCCTCAGCAGGAAAC<br>AGCCTGCTGCACACTGGATAC
FBLN4	NM_016938.3	141	64	3.0	ACAACCGCTCCTGTGTTGATGTG<br>TATCACTGCAGGAGAAGCCATCC
FBLN5	NM_006329.4	99	61	3.5	CGCTGTGACCCAGGATATGAAC

					CACACACTCATGTTGGCAGAGG
FBLN6	NM_031935.3	189	63	3.5	TGGTTGCATACACACAGGATGGAG<br>TCCCATTGGCACTGTAGGATGAGG
FBLN7	NM_153214.2	245	64	3.5	GAAGAGCTGTGAGGATGTGGATG<br>TGGAAGGCAGAGAGAGGTAATGG
FBN1	NM_000138.5	184	64	3.5	GAATGCAAGAACCTCATTGGCAC<br>TGGCGGTAAACCCATCATTACAC
FN1	NM_054034.3	205	62	3.5	GATGTTCGTGCAGCTGTTTACC<br>TTGAGTTGCCACCGTAAGTCTG
ISLR2	NM_020851.3	119	62	3.0	CGCTGTCACACTTGCAACTC<br>ACAAGCAATGGAGTCGGGC
GAPDH	NM_002046.6	194	64	3.0	AAGGTCGGAGTCAACGGATTTGG<br>ATGACAAGCTTCCCGTTCTCAGC
HPRT1	NM_000194.3	229	58	3.5	TGCTGACCTGCTGGATTACA<br>CCTGACCAAGGAAAGCAAAG
HSD17B2	NM_002153.3	163	62	3.0	AAGGGAGGCTGGTGAATGTCAG<br>AAGCCTCCAGGTTGGATGGAAG
LOX	NM_002317.3	104	62	3.5	CAGATTTCTTACCCAGCCGACC<br>GGCATCAAGCAGGTCATAGTGG
LOXL1	NM_005576.2	142	62	3.5	ACTACGATGTGCGGGTGCTACTG<br>TGGCTGAACTCGTCCATGCTGTG
LTBP1	NM_000627.4	140	65	3.0	TGAGGAGTTACAGGCTGAGGAATG<br>TTACATCGACACAGGTCATCTTGG
LTBP2	NM_000428.3	129	65	3.0	TGAGAAGGGCTGCCAAGATGTGG<br>CCGTCTTCATTCACCCAGTACCC
LRAT	NM_004744.5	135	61	3.0	ACTGCGAGCACTTCGTGACC<br>ACGCCAATCCCAAGACTGCT
MFAP2	NM_017459.3	179	62	3.0	CCAGCAGCAAGTCCAACAGGAAG<br>GGCTGTAGAAGCAGACCTCGTTG
MMP2	NM_004530.6	179	63	3.5	CTCTCCACTGCCTTCGATACACC<br>CTGGGAGGAGTACAGTCAGCATC
NR1H2	NM_007121.7	189	63	3.0	GCGCTACAACCACGAGACAGAG<br>GCCGAGAAGATGTTGATGGCGA
NR1H3	NM_005693.4	186	63	3.0	CGAGACTTCTGGACAGGAAACTG<br>CACCGCAGAGTCAGGAGGAATG
OTX2	NM_172337.3	161	62	3.5	AGTCTGACCACTTCGGGTATGG<br>TCTCGCATGAAGATGTCTGGGT
PLAT	NM_033011.4	192	62	3.5	TGTCAGACTGTACCCATCCAGC<br>ATGATGCCCACCAAAGTCATGC
PPARA	NM_005036.6	130	62	3.0	AGGAGACTGAACTGACCACAT<br>ACCATTAAGGCAAAACTGTACCCT
PPARG	NM_138712.5	90	64	3.0	ACTTGTACTAGCAGAGAGTCCTGA<br>ATTTCTTAGGTGTCAGATTTTCCCT
PPARD	NM_006238.5	181	61	3.0	CATGCCCCAGGATCACTCTC<br>GTATCCTCAGCACCAGACCC
RARA	NM_000964.3	242	63	3.0	GGGGGAATCCTGAATCGAGCTG<br>GGTTGGGGGATGTCTCAAGAGC
RARB	NM_000965.5	230	61	3.0	AACGTCTGCCTGGTTTCACTGG<br>TTCCAAAGGCAGGAGCTGGTTG
RARG	NM_000966.6	152	61	3.0	TCGCCGAAGCATCCAGAAGAAC<br>TCCGGTCATTTCGCACAGCTTC

RBP1	NM_002899.5	148	59	3.0	CTCCTTCTCACCCTTCTGCA<br>CTCCAGTCACTCCCCGAAAT
RBP2	NM_004164.2	247	59	3.0	TGGAACCTGGGAGATGGAGAGT<br>GGTGACCAGTGCCTTAACATGC
RBP3	NM_002900.2	205	63	3.0	CATGGGCACCTCCGCCTTAG<br>CACATCCTTGTCGGCACCGT
RBP4	NM_006744.4	165	59	3.0	ACACGAAGGAGTAGCTGTCA<br>ACCCTGCCAAGTTCAAGATG
RDH5	NM_001199771.1	138	63	3.0	CCAATGGTGGGGGCTACTGTG<br>CCAGGTTGGTCACAGGGGTTC
RDH10	NM_172037.5	165	63	3.0	ATGGTGCGCCACATCTACCG<br>GCGGACTCTTTCAGCCGTCA
RORA	NM_134261.3	177	62	3.0	AATGCCTTGCCGTAGGGATGT<br>TGGCCGAGATGTTGTAGGTGG
RORB	NM_006914.4	195	59	3.0	AAGGGATGGTTTTCTCGGCAG<br>GATCCCAGAGGACTTATCGCCA
RORC	NM_005060.4	154	59	3.0	TGGCTGCAAAGAAGACCCACAC<br>CAGGAGTAGGCCGCGTTACA
RXRA	NM_002957.5	178	62	3.0	GAGTTAGTCGCAGACATGGACAC<br>TCAGGGTGCTGATGGGAGAATG
RXRB	NM_021976.5	178	61	3.0	CTGCCGCTATCAGAAGTGCCTG<br>GCCCTGGTCACTCTTCTGTTCC
RXRG	NM_006917.5	205	61	3.0	CAGCTCTCAGCTAAATGTGGTC<br>CTTGAAGAACCCTTTGCAGCCT
SOX2	NM_003106.4	131	60	3.0	GAAGGATAAGTACACGCTGCCC<br>CGTTCATGTGCGCGTAACTGTC
SREBF1	NM_004176.5	175	63	3.0	GTGACCTCGCAGATCCAGCAG<br>CACTCACCAGGGTCGGCAAAG
STRA6	NM_022369.4	119	59	3.0	TTGGAGCCTCAGTTTTCCCA<br>TGCTGCTGTTTTCATGGTCC
TGFB1	NM_000660.7	121	60	3.0	CTGCGGATCTCTGTGTCATTGG<br>CGGGCAAAGGAATAGTGCAGAC
TGFBR2	NM_003242.6	174	62	3.0	TCGGTCTATGACGAGCAGCG<br>GTGGAAACTTGACTGCACCGTT
TIMP2	NM_003255.4	117	63	3.5	TGTTCCCTCCCTCAAAGACTGAC<br>CCACCTACCTCCAAAATCCTTCC
TTR	NM_000371.3	108	62	3.0	TGTCTGAGGCTGGCCCTACG<br>ACACATGCACGGCCACATTG
WNT8A	NM_058244.4	150	61	3.0	TCTTCAGCTCTCCACCCACAAC<br>TTGTTTGACCCATCACAGCCAC

Table S4. Primary antibodies

Antibody (clone), Host species	Antibody concentration	Application	Antibody source
Actin beta (AC-15), mouse	1:10000	Western Blotting	Sigma-Aldrich
Elastin (B-10), mouse	1:250	Western Blotting	Santa Cruz
Fibrillin-1 (MAB 26), mouse	1:2500	Western Blotting	Merck/Millipore
LOXL1, rabbit	1:1000	Western Blotting	LSBio
Phospho-Smad2 (138D4), rabbit	1:1000	Western Blotting	Cell Signaling
RARα (C-20), rabbit	1:2500	Western Blotting	Santa Cruz
Smad2 (L16D3), mouse	1:1000	Western Blotting	Cell Signaling
Fibrillin-1, rabbit	1:2000	Immunocytochemistry	Dieter Reinhardt
LOXL1, mouse (B01P)	1:50	Immunohisto/cytochemistry	Abnova
LOXL1, rabbit	1:1000	Immunohisto/cytochemistry	Takako Sasaki
LTBP-1, mouse (H-1)	1:50	Immunocytochemistry	Santa Cruz
RARα (C-20), rabbit	1:500	Immunohistochemistry	Santa Cruz
RXRα (4E9), mouse	1:50	Immunohistochemistry	Merck/Millipore
RXRα (EPR7106), rabbit	1:200	Immunohistochemistry	Abcam
Smad2/3 (D7G7), rabbit	1:300	Immunohisto/cytochemistry	Cell Signaling
Tropoelastin, rabbit	1:200	Immunocytochemistry	EPC
Phalloidin, Alexa Fluor 555	1:50	Immunocytochemistry	Cell Signaling

Figure S1: Comparative analysis of retinoic acid pathway components in ocular tissues of normal and PEX eyes with different stages of disease. Relative expression levels of transcripts for STRA6 (signaling receptor and transporter of retinol 6), RBP1 (retinol binding protein 1), ADH1 (alcohol dehydrogenase 1), ALDH1A1 (aldehyde dehydrogenase 1 A1) and RARA (retinoic acid receptor alpha) in ciliary body tissues obtained from normal human donors as control (n=23) and patients with early stages (n=7) and late stages (n=17) of PEX syndrome analyzed by real-time PCR. Expression levels were consistently reduced in all disease stages including early stages. Expression levels were normalized relative to GAPDH and HPRT1. Data are given as fold change of expression in PEX as compared to control samples set to 1 (*p<0.05, **p<0.01, ***p<0.001).

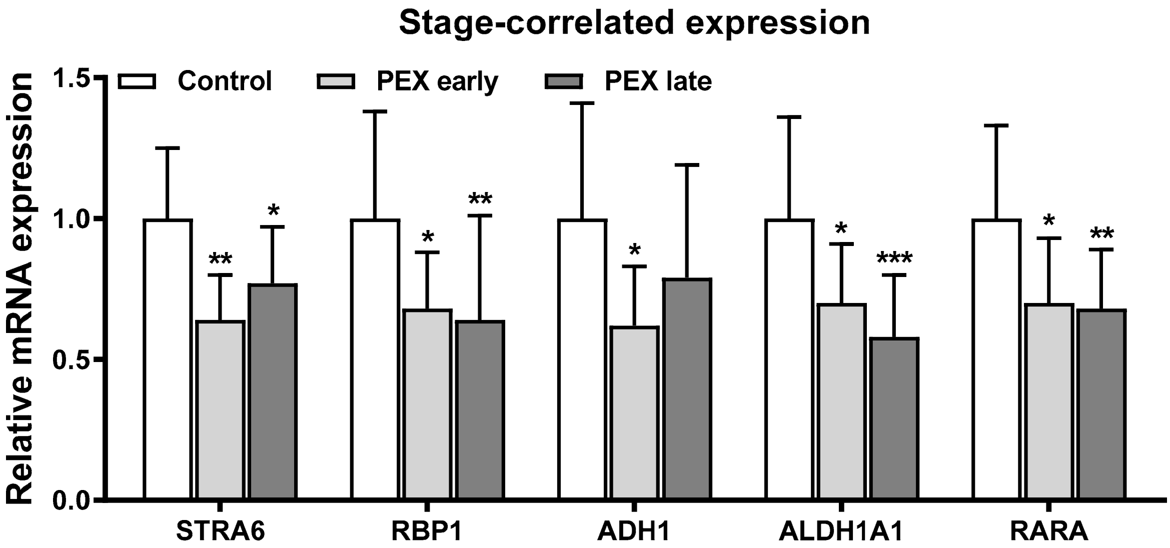

Figure S2: Correlation of RBP3 (retinol-binding protein 3), RBP4 (retinol-binding protein 4) and TTR (transthyretin) levels and total protein concentrations in aqueous humor samples of PEX and control eyes.

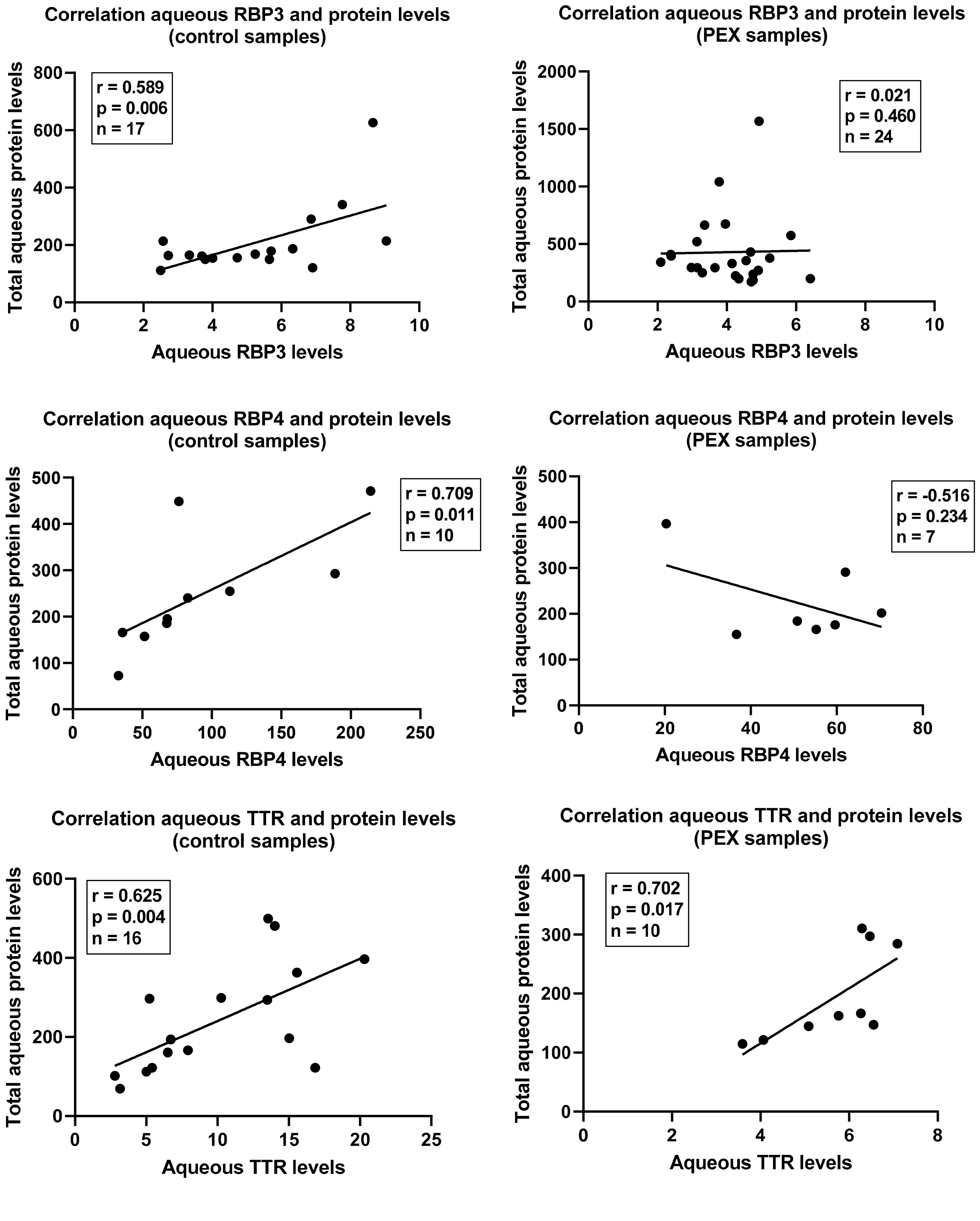

Figure S3. Dose-response effects of retinoic acid signaling activation by all-trans retinoic acid. Real-time PCR analysis of LOXL1 (lysyl oxidase-like 1), ELN (elastin) and FBN1 (fibrillin-1) expression in cultured human Tenon's capsule fibroblasts (hTCF) without stimulation (control, Ctrl) and in response to various concentration of all-*trans* retinoic acid (ATRA) (n=4). Expression levels were normalized relative to GAPDH and HPRT1. Data are given as fold change of expression in PEX as compared to untreated control samples set to 1 (*p<0.05, **p<0.01, ***p<0.001).

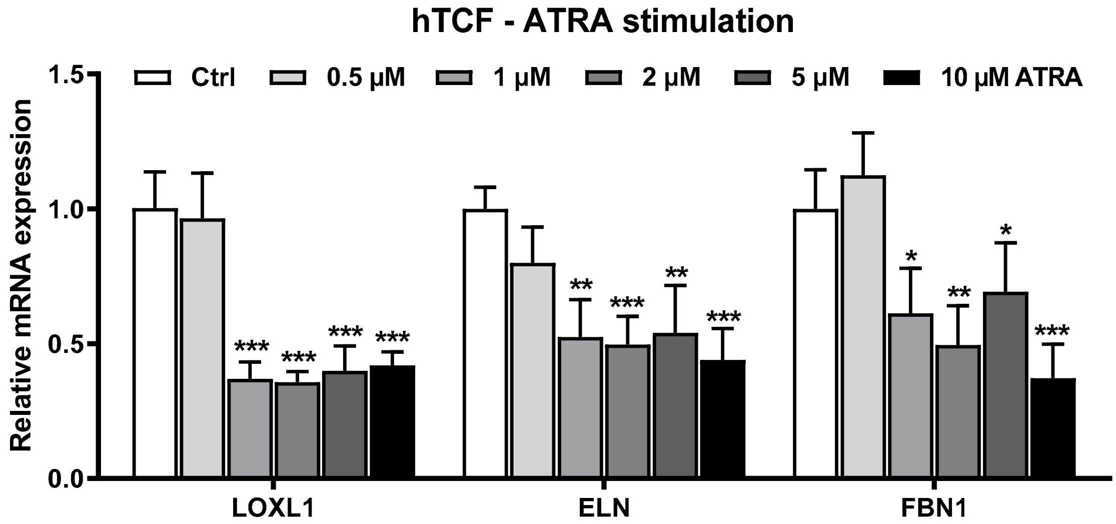

Figure S4. Dose-response effects of retinoic acid signaling activation by synthetic retinoids. Real-time PCR analysis of LOXL1 (lysyl oxidase-like 1), ELN (elastin), FBN1 (fibrillin-1) and LTBP1 (latent transforming growth factor beta binding protein 1) expression in cultured human Tenon's capsule fibroblasts without stimulation (control, Ctrl) and in response to 2 µM all-*trans* retinoic acid (ATRA, pan-RAR agonist), 0.01-10 µM AM 80 (RARα agonist), AC 261066 (RARβ agonist), CD 1530 (RARγ agonist), LGD 1069 (pan-RXR agonist) and CD 3254 (RXRα agonist) (n=4). Expression levels were normalized relative to GAPDH and HPRT1. Data are given as fold change of expression in PEX as compared to untreated control samples set to 1 (*p<0.05, **p<0.01, ***p<0.001).

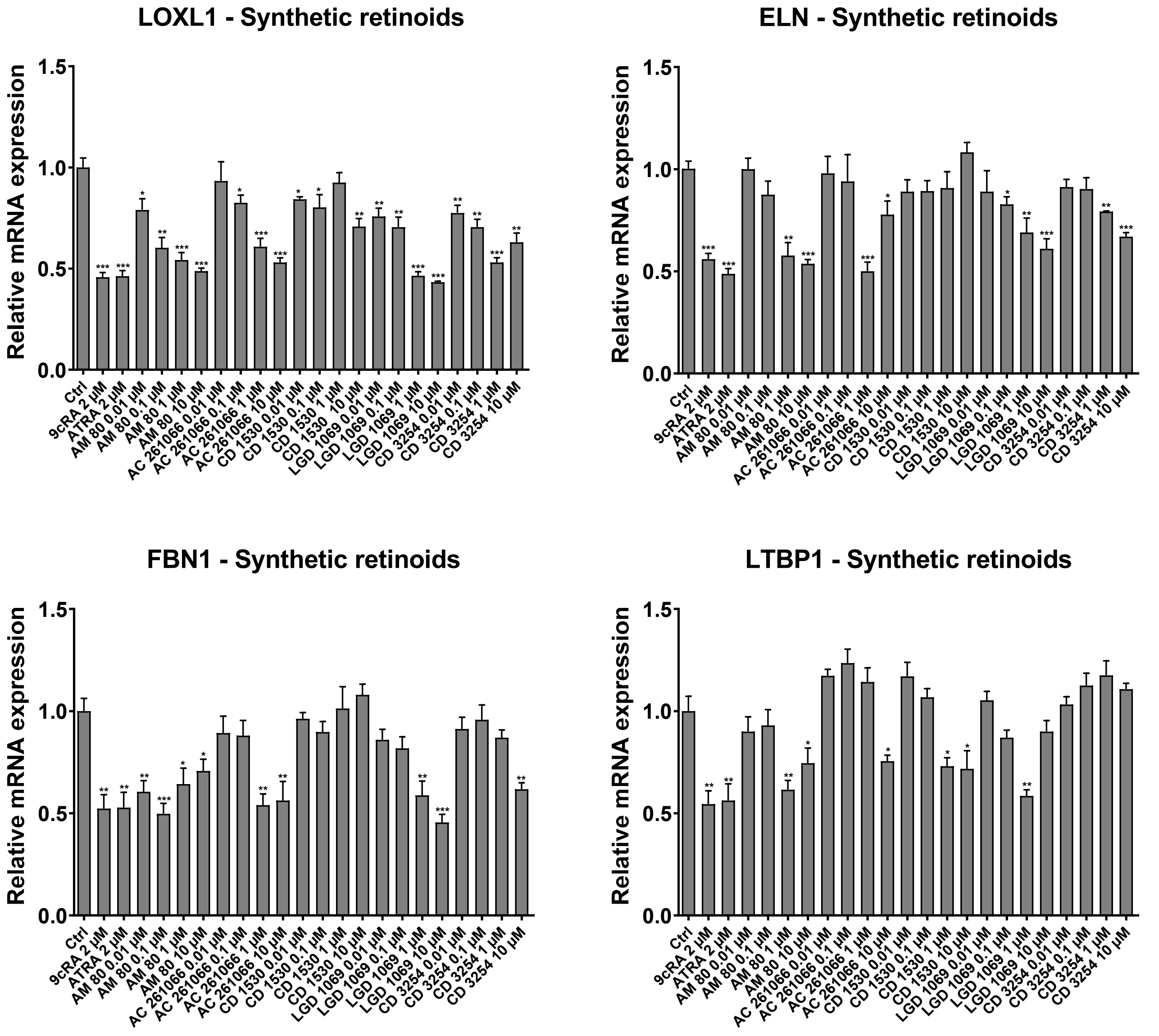

Supplement: Supplementary file 1 [file ijms-23-05977-s001.zip › ijms-1742846-supplementary.pdf]
